# The Impact of Liquor Restrictions on Serious Assaults across Queensland, Australia

**DOI:** 10.3390/ijerph16224362

**Published:** 2019-11-08

**Authors:** Nicholas Taylor, Kerri Coomber, Richelle Mayshak, Renee Zahnow, Jason Ferris, Peter Miller

**Affiliations:** 1 School of Psychology, Deakin University, Geelong Waterfront Campus, Geelong, VIC 3220, Australia; k.coomber@deakin.edu.au (K.C.); richelle.mayshak@deakin.edu.au (R.M.); peter.miller@deakin.edu.au (P.M.); 2 School of Social Science, University of Queensland, St Lucia, QLD 4072, Australia; r.zahnow@uq.edu.au; 3 Centre for Health Services Research, University of Queensland, St Lucia, QLD 4072, Australia; j.ferris@uq.edu.au

**Keywords:** nightlife, night-time economy, alcohol drinking, policy, alcoholic beverages, assault

## Abstract

*Aims*: This study aimed to explore the relationship between a 00:00 liquor restriction, introduced on 1 July 2016, and alcohol-related harm by examining its impact on serious assault numbers during high-alcohol hours (20:00–6:00 Friday and Saturday night), from 1 January 2009 to 30 June 2018. *Methods*: Two types of locations only impacted by the liquor restriction were identified: designated safe night precincts (SNPs) and other local government areas (LGAs). A times series autoregressive integrated moving average analysis was used to estimate the influence of liquor restrictions on police-recorded serious assaults in the two years following the policy introduction, for SNPs and LGAs separately. *Results*: Contrarily to our predictions, monthly police-recorded serious assaults did not significantly change within SNPs or LGAs following the introduction of liquor restrictions. *Conclusion*: The implementation of the Queensland liquor restriction did not result in a clear, unique reduction in serious assault trends. Further investigation should consider the impact of liquor restrictions in conjunction with other policy changes as public perception of restrictions and their cumulative impact may produce varied outcomes.

## 1. Introduction

Alcohol-related harm in night-time entertainment precincts (NEPs) is a major preventable burden on the community [1]. NEPs are high-risk areas defined by a cluster of on-licence venues, including pubs, bars, and nightclubs [2]. These areas are often identified as ‘hotspots’ for violence [3,4,5] due to high levels of intoxication [6] and an atmosphere of permissiveness and anonymity [7]. Much of the experiences of harm in NEPs can be attributed to risky alcohol consumption patterns [8], particularly among heavy alcohol consumers and those who become acutely intoxicated by consuming liquor with a high alcohol content [8,9,10]. Many interventions have tried to directly target consumption of various ‘high-risk liquors’ (e.g., shots, mixed liquor with more than 30 mL of alcohol, ready mixed liquor with over five percent alcohol, or over four alcoholic beverages to a single patron past 22:00; [11]). However, empirical evidence of the effectiveness of these alcohol restrictions is lacking [12]. One particular alcohol restriction that has become popular among policy makers despite a lack of empirical evidence [12] is the ban of shots after a certain time of night (e.g., 22:00 [11,13], 00:00 [14,15], 1:00 [16], or 4:00 [17]). The popularity of this approach is likely owing to its simplicity in both implementation [11], and its apparent face validity, as it gives the appearance of addressing the problem [12]. 

Previously, liquor restrictions have been introduced as one part of a package of interventions rather than on their own [12,18,19]. Because of this, it has been difficult to interpret the unique impact of the restrictions, or to determine whether it is independently effective at reducing alcohol-related harm. In Newcastle and Sydney, ‘package’ policy changes that included liquor restrictions were followed by a decrease in assaults and serious emergency department injury presentations, respectively [13,15]. Much of this impact was attributed to other simultaneously introduced restrictions on the availability of alcohol, such as changes in trading hours in Newcastle [13]. The Sydney study did not attribute change in harms to any one component of a legislative package, as separate effects could not be determined by the method of measurement used [15]. Despite the lack of clear evidence in the literature, a consensus of expert opinions concluded that there was moderate support for the use of restrictions on the sale of shots and similar liquor from 22:00 for reducing alcohol consumption, intoxication, alcohol-related assaults, alcohol-related harm, and crime [11]. 

On 1 July 2016, liquor restrictions were introduced across the state of Queensland, Australia, as a part of the ‘Tackling Alcohol-Fuelled Violence Legislation Act’ (TAFV; [20]). Specifically, the sale of liquor that is designed to be consumed rapidly or contains a high percentage of alcohol was banned after 00:00 [20]. Exemptions to this regulation can be granted so long as the venue primarily sells high value or quality spirits and seats 60 or less patrons [20]. These restrictions were implemented as one part of a package of interventions aimed at reducing alcohol-fuelled violence, including a trading hours restriction [20]. Fifteen prominent NEPs designated by the government as ‘Safe Night Precincts’ (SNPs) were given a 3:00 trading hours restriction, reduced from a previous 5:00 close, while the rest of the state were required to adhere to a 2:00 trading hours restriction, rolled back from a 3:00 close [20]. A restriction on the use of extended trading permits was also introduced in February 2017 and mandatory identification scanners were introduced to SNPs in July 2017. 

The policy was followed by a reduction in serious late-night assaults in Queensland’s largest SNP [21]. However, the introduction of liquor sales restrictions alongside a trading hours restriction limits the ability for researchers to evaluate the unique impact of each intervention. Importantly, any decrease in alcohol-related harm due to liquor restrictions would likely be masked due to the well-established and large impact trading hours restrictions have on alcohol-related harm in NEPs [12]. As such, any change in alcohol-related harm in an area where both liquor and trading hours restrictions were implemented would likely be attributed to changes in trading hours. However, there were conditions specific to the implementation of the Queensland policy that permit the examination of the unique impact of liquor restrictions for the first time. There were SNPs with licenced venues that did not trade later than 3:00 *before* the new regulations were introduced. As such, these venues did not experience any change in trading hours as a result of policy implementation. The only change experienced by these venues was the restriction on rapid intoxication liquor after 00:00. In addition, as the introduction of the trading hours and liquor restrictions was statewide, the sample included Queensland local government areas (LGAs) with no SNPs or late-trading venues. Venues in these LGAs were only directly engaged in the liquor restrictions element of the state liquor policy and did not experience changes in trading hours. These areas serve as sites to examine the unique implications of liquor restrictions when introduced as a solitary alcohol-control measure.

Liquor restrictions are becoming a part of the night-time environment, yet there is limited evidence to guide researchers and policy makers about their role in reducing alcohol-related harm [12]. This study aimed to explore the relationship between a 00:00 liquor restriction and alcohol-related harm by examining its impact on serious assault numbers during high-alcohol hours (HAH, 20:00–6:00 Friday and Saturday night; [22]) in the two years following the introduction of the TAFV policy. The use of a HAH proxy measure was chosen despite police recording alcohol involvement, as these assessments are often inaccurate or made inconsistently [23,24,25,26]. It was hypothesised that

1. Police-recorded serious assaults per month during HAH will significantly decrease after the introduction of liquor restrictions in SNPs where trading hours restrictions did not alter any venues’ trading hours, particularly after 00:00 when the liquor restrictions take effect.
2. Police-recorded serious assaults per month during HAH will significantly decrease after the introduction of liquor restrictions in LGAs with no SNP where trading hours restrictions did not alter any venues’ trading hours, particularly after 00:00 when the liquor restrictions take effect.

## 2. Method

### 2.1. Data

The data used in this study were obtained from the Queensland Alcohol-related violence and Night Time Economy Monitoring project (QUANTEM; [14]). Ethics approval was provided by the Human Research Ethics Committees of Deakin University, The University of Queensland, and James Cook University. Information on all venues licensed to sell liquor in Queensland, including licence number, licence type, licensed hours of operation, approved extended trading permits, and address, was provided by the Queensland Governments’ Office of Liquor Gaming and Regulation. De-identified unit records of police recorded assaults, including specifications about the type of assault, were obtained from the Queensland Police Service. Records of serious assault that resulted in bodily harm were analysed, which included assaults resulting in bodily harm, aggravated non-sexual assault, assault that results in bodily fluid entering the victim, serious assault (other), grievous bodily harm, and wounding [14]. Night-time serious assaults were used as they are considered a reliable indicator of alcohol-related harm, unlike other police-recorded incidents which are more likely to be influenced by police activity and enforcement strategies [27]. Assaults within each SNP and LGA during HAH from 1 January 2009 to 30 June 2018 were included in the analysis. Assaults that occurred on residential property were excluded from analyses. 

### 2.2. Setting

Using licencing data from 30 June 2016, five SNPs were identified where no venues were affected by the 3:00 trading hour restriction. Table 1 shows details of the venues in each SNP as well as the number of venues that were licenced to serve past midnight, and therefore affected by the liquor restrictions. 

Using licencing data provided from 30 June 2016, 15 LGAs were identified where no venues were affected by the 2:00 trading hour restriction and at least one venue was impacted by the 00:00 liquor restriction. Table 2 shows details of the venues in each LGA as well as the number of venues that were licenced to serve past midnight, and therefore, affected by the liquor restrictions. 

### 2.3. Analysis

All SNPs and LGAs were aggregated into two categories for analysis; SNPs with venues only affected by liquor restrictions, and LGAs with venues only affected by liquor restrictions. Times series autoregressive integrated moving average (ARIMA) analysis [28] was used to estimate the influence of liquor restrictions on monthly police-recorded assaults for each category. A time series approach was used as it allows for the assessment of the acute and gradual effects of an intervention on trends across time [28]. The analysis allows for the examination of long-term trends, rather than just providing a pre-post comparison. The data used are particularly suited to this approach, and the method is commonly used to assess the impact of interventions on epidemiological outcomes (e.g., 21). The standard modelling approach for ARIMA analyses was used [28]. Where data exhibited clear positive or negative trends, first order differencing was used to transform the data into a stationary series. After differencing the data series, seasonality was assessed by examining the autocorrelation and partial autocorrelation plots; seasonal models were fitted where periodic trends were observed (e.g., a spike every 6 months; [29]). The auto-regressive and moving average values were determined by examining the autocorrelation plots and partial autocorrelation plots, respectively, and using reference plots to assign these terms to the ARIMA models [30]. Cross-correlograms were examined to identify the best-fitting transfer function for the intervention variable (specified as lag; [29]). The introduction of the liquor restriction policy was coded as a step function, as it was introduced abruptly and remained until the end of the period analysed [30]. All analyses were undertaken using Stata 15.0 (StataCorp LLC, College Station, TX, USA) [31].

## 3. Results

### 3.1. SNPs with Venues Only Affected by Liquor Restrictions 

**Figure 1:**
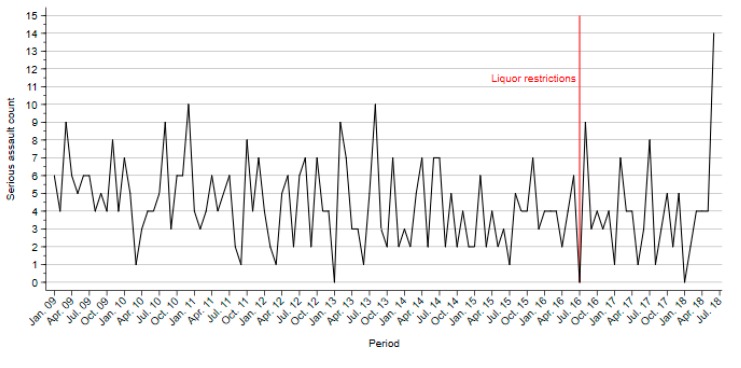
Serious assaults in safe night precincts with venues only affected by liquor restrictions during high-alcohol hours.

No seasonality was found in the model. The analysis found no significant change in the number of serious assaults per month after the introduction of liquor restrictions during HAH in SNPs that were not affected by restrictions on trading hours (ARIMA(0,0,1), *Q* = 30.93, *p* = 0.85; Figure 1). June 2018 saw a large rise in serious assaults; however, removing this outlier from the analysis did not alter the finding. 

### 3.2. LGAs with Venues Only Affected by Liquor Restrictions

**Figure 2:**
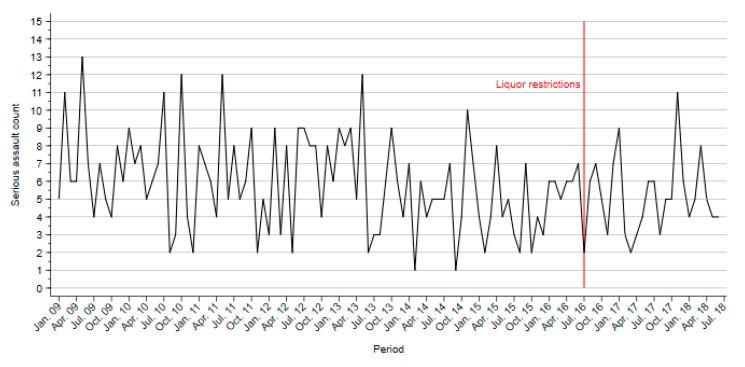
Serious assaults in local government areas with venues only affected by liquor restrictions during high-alcohol hours.

No seasonality was found in the model. The analysis found no significant change in the number of serious assaults per month after the introduction of liquor restrictions during HAH in LGAs that were not affected by restrictions on trading hours (ARIMA(0,1,1), *Q* = 29.84, *p* = 0.88; Figure 2).

### 3.3. Sensitivity Analyses 

HAH range from 20:00 to 6:00; however, liquor restrictions were only active from 00:00. Because of this, additional analyses were run across all sites to examine whether serious assaults decreased between midnight and 6:00. The ARIMA model specifications were the same for the HAH models and the 00:00–6:00 models. No seasonality was found in either model. No significant changes in monthly serious assault trends (*p* > 0.05) were found in either the SNP or LGA models. 

Mandatory identification scanners were introduced into all SNPs in July 2017. Further analyses were run in order to control for the introduction of the additional intervention in the SNP models, this was coded as an additional step function. However, this did not contribute significantly to either the full HAH model, or the 00:00–6:00 model.

## 4. Discussion 

This study aimed to examine the impact of a midnight liquor restriction on the number of serious assaults per month in nightlife spaces across Queensland. The intervention was introduced as part of a ‘package’ of interventions, notably alongside a trading hours restriction. There were two areas where the impact of the liquor restrictions could be separated from the trading hours restriction: SNPs where all venues closed before 3:00, and LGAs where all venues closed before 2:00. The hypotheses were not supported as the number of serious assaults per month did not significantly change in either area analysed. 

### 4.1. Liquor Restrictions in SNPs

The first hypothesis predicted that there would be a significant decline in the number of serious assaults per month after the introduction of liquor restrictions in SNPs during HAH, especially after 00:00. There was no significant change in serious assaults observed during HAH or past midnight. While other interventions were introduced into SNPs, controlling for these did not meaningfully contribute to the model. One of the main focuses of the TAFV policy was to reduce alcohol-fuelled violence within SNPs [20], meaning that if the liquor restriction had an impact it should have been strongest in these areas. The lack of any significant change suggests that liquor restrictions may not be an effective standalone intervention within the context of NEPs. However, many SNPs could not be included in the current study due to the simultaneous introduction of a trading hours restriction. The SNPs included in the current analysis were predominately composed of hotels, and it is possible that the liquor restriction had a different impact across venue types. However, assaults could not be distinguished by venue type because the close proximity of venues in SNPs prevents association of an assault with a specific venue. To highlight the extent of the problem within SNPs it should be noted that these serious assaults all occurred within a concentrated geographical space. SNPs included in the analysis totalled to 3.8 km^2^ with 130.53 serious assaults per square kilometre across the period analysed, in comparison to LGAs without a SNP at 134,463.7km^2^ with <0.01 serious assaults per square kilometre across the period analysed. 

### 4.2. Liquor Restrictions in LGAs with no SNP

The second hypothesis predicted that there would be a significant decline in the number of serious assaults per month after the introduction of the liquor restrictions in LGAs without an SNP during HAH and after 00:00. There was no significant change in serious assaults observed during HAH or past midnight. As with the SNPs analysed, the LGAs included in the analysis were predominantly comprised of hotels. The areas included in these analyses were mostly small towns, with nightlife spaces much smaller than what would be considered a NEP [2]. However, the liquor restrictions still applied to these areas, and there is evidence to suggest restrictions on ‘high-risk liquor’ have reduced violence in small communities previously [32,33]. Liquor restrictions also only operated for a maximum of two hours in these areas, which was expected to be less impactful than the three-hour restriction in SNPs. The current results suggest that the alcohol-related assaults experienced in these communities are unlikely to be due to the on-licence consumption of ‘high-risk liquor’ beyond midnight.

### 4.3. Implications for Future Interventions 

There are multiple theoretical explanations as to why this intervention had no observable impact, which can be built upon by future interventions. As the liquor restrictions are only in effect after midnight, they would not be completely effective against individuals who are already heavily intoxicated by this time, or who are aware of the regulation and are able change their alcohol consumption patterns in response to it. This differs from a comparable intervention on another form of ‘high-risk liquor’, namely alcopops [34]. In April 2008, the Australian government imposed a 70% nationwide tax increase on these beverages, which was associated with significant decreases in emergency department presentations for acute alcohol problems [34]. This price-based intervention reduced the economic availability of the ‘high-risk liquor’ across the entire day; notably, all previous effective restrictions on ‘high-risk liquor’ restricted access to the liquor across the entire day [32,34]. However, this is not true of liquor restrictions within NEPs. 

The hypotheses of the current study were partially based on research indicating acute consumption of alcohol may lead to increased experiences of alcohol-related harm, although the literature these hypotheses were based on used survey or observational data and did not measure the impact of a trial [6,8]. There is also no evidence available to suggest that liquor restrictions affect this consumption pattern. Even if liquor restrictions do target this form of alcohol consumption, it is possible that it simply misses the mark on how heavy intoxication, and in turn increased experiences of alcohol-related harm, occurs in nightlife spaces. The current restriction targeted highly concentrated, low value, low quality liquor that could be consumed quickly beyond midnight (e.g. shots; [20]). However, many studies have suggested that it is patrons who enter nightlife spaces already intoxicated that are the most likely to experience alcohol-related aggression [35,36]. Self-report measures indicate that this is a stronger predictor of violence than the amount or type of alcohol consumed on a night out [35,36]. These individuals are likely to be more heavily intoxicated sooner and for longer periods of the night, which increases the likelihood of aggressive incidents occurring [35]. 

The liquor restrictions in Queensland allowed for exemptions for venues that seated 60 or fewer patrons to still serve high quality or value liquor [20], meaning potentially risky beverages could still be served past midnight in each of the areas examined. While previous support for liquor restrictions has focused on bans that take effect from 22:00 [11] there is no direct empirical evidence to suggest that this would be more effective [12]. This highlights a key issue with the implementation of shot bans, which is the seemingly arbitrary designation of hours after which all shots are excluded, ranging from as early as 22:00 [11,13] to as late as 4:00 [17]. This issue undermines the widespread use of the policy, as there is no consistent and clear theoretical reasoning for its implementation [12], which is a core component of effective alcohol policy [37]. 

### 4.4. Limitations 

Due to the concurrent implementation of the trading hours restriction the current study could not determine the impact of a 00:00 liquor restriction on areas where alcohol was previously available past 3:00. These areas include the largest NEPs in Queensland, experience the highest amount of assaults and have a greater diversity of venue types than the precincts examined; as such their exclusion was a limitation of the study. Covariates such as the characteristics of individuals involved in each serious assault and their consumption patterns were beyond the scope of this study. The study was also unable to determine whether patron behaviour changed in an attempt to undermine the liquor restriction (e.g., stockpiling high-risk liquor before midnight). 

## 5. Conclusions 

Liquor restrictions are a popular policy measure that have been implemented across various NEPs for over a decade [12]. This evaluation was the first to examine the unique impact of liquor restrictions in isolation from ‘package’ alcohol intervention policies. The results of this study found no significant effect of these liquor restrictions in reducing alcohol-related harms in NEPs, specifically in the form of police-recorded assaults. Nonetheless, this single study has a range of relevant limitations which mean that definitive statements about effectiveness in different jurisdictions are unwise. Ideally, a liquor restriction intervention would be trialled with control conditions in other jurisdictions and with limits on the other interventions in place. Further investigation should also consider the impact of liquor restrictions in conjunction with other policy changes as public perception of restrictions and their cumulative impact may produce varied outcomes. Regardless, the current study suggests, in line with a wide range of previous theory and evidence, that liquor restrictions as a stand-alone measure in nightlife settings are unlikely to be associated with significant reductions in serious assaults.

## Figures and Tables

**Table 1:** Venue details for safe night precincts (SNPs) with closing times after 00:00 and not after 3:00 before July 2016.

SNP	N Licences	High-Risk Venue Types			Venues Affected by Liquor Restriction
		Bar	Hotel	Nightclub	
Bundaberg CBD	26	0	7	0	7 *
Caloundra	49	0	3	0	3
Ipswich CBD	26	0	12	0	1
Maroochydore	39	1	3	2	6 *
Mooloolaba	55	0	3	0	4 *

*Note*: SNP = Safe-night precinct; CBD = Central Business District, * Totals include low risk venues that were also affected: one community club in Bundaberg, one in Maroochydore, and one restaurant in Mooloolaba.

**Table 2:** Venue details for LGAs with closing times after 00:00 and not after 2:00 before July 2016.

LGA	N Licences	High-Risk Venue Types			Venues Affected by Liquor Restriction
		Bar	Hotel	Nightclub	
Barcaldine	19	0	10	0	1
Charters Towers	35	0	17	0	2
Cloncurry	23	0	8	0	4
Diamantina	4	0	2	0	1
Flinders	8	0	4	0	1
Goondiwindi	35	0	10	0	4
Isaac	63	0	11	0	2
Lockyer Valley	47	0	17	0	3
Longreach	24	0	8	0	2
Scenic Rim	122	0	21	0	3
South Burnett	92	0	24	0	4 *
Southern Downs	159	1	22	0	3 *
Tablelands	60	0	16	0	3 *
Weipa	8	0	1	0	1 *
Winton	10	0	5	0	1

*Note:* LGA = local government area, * Totals include low risk venues that were also affected: one community club in South Burnett, one in Southern Downs, one in Tablelands, and one in Weipa.
